# Fish Oil Feeding Modulates the Expression of Hepatic MicroRNAs in a Western-Style Diet-Induced Nonalcoholic Fatty Liver Disease Rat Model

**DOI:** 10.1155/2017/2503847

**Published:** 2017-06-12

**Authors:** Hualin Wang, Yang Shao, Fahu Yuan, Han Feng, Na Li, Hongyu Zhang, Chaodong Wu, Zhiguo Liu

**Affiliations:** ^1^School of Biology and Pharmaceutical Engineering, Wuhan Polytechnic University, Wuhan, Hubei 430023, China; ^2^School of Medicine, Jianghan University, Wuhan, Hubei 430000, China; ^3^Department of Nutrition and Food Science, Texas A&M University, College Station, TX 77843, USA

## Abstract

Nonalcoholic fatty liver disease (NAFLD) is one of the most prevalent chronic liver diseases worldwide. Recent studies have indicated that fish oil supplementation has benefits against NAFLD. Our previous transcriptomic study has validated the effect of fish oil supplementation on altering hepatic gene expression in a NAFLD rat model. In the current study, we examined the effects of fish oil on the expression of hepatic microRNAs. Male Sprague–Dawley rats were fed with a lab chow (CON), high-fat high-cholesterol diet (WD), or WD supplemented with fish oil (FOH), respectively. Small RNAs were extracted from livers for RNA-sequencing. A total of 79 miRNAs were identified as differentially expressed miRNAs (DEMs) between FOH and WD groups, exemplified by rno-miR-29c-3p, rno-miR-30d-5p, rno-miR-33-5p, rno-miR-34a, and rno-miR-328a-3p. Functional annotation of DEMs predicted target genes suggested that the altered hepatic miRNAs contributed to fish oil modification of hepatic lipid metabolism and signaling transduction. Integrative analysis of DEMs and differentially expressed genes suggested that the expression difference of* Pcsk9, Insig2, Per3,* and* Socs1/3* between FOH and WD groups may be due to miRNA modification. Our study reveals that fish oil supplementation alters hepatic expression of miRNAs, which may contribute to fish oil amelioration of NAFLD in rats.

## 1. Introduction

Over the past four decades, overweight and obesity have been implicated as a severe public health problem worldwide. Also, global prevalence of obesity has kept increasing quickly since 2000 [1]. The spread of Western lifestyle, such as chronic and excessive consumption of the “Western-style” high-fat diets, was considered as a major causal factor of the epidemic of obesity and related metabolic disorders, such as hyperlipidemia, hypertension, and insulin resistance [2]. During obesity, excessive fat intake enhances liver uptake of fatty acids and glycerol (substrates of triglycerides) from the circulation. Additionally, obesity-associated insulin resistance and hyperinsulinemia result in increased hepatic de novo lipogenesis [3, 4]. When having inefficiency in removing lipids through beta-oxidation or efflux, the liver is overloaded with triglycerides in the form of lipid droplets in hepatocytes, leading to hepatic steatosis. The latter is the initial stage of nonalcoholic fatty liver disease (NAFLD) [5, 6]. Ectopic deposition of lipids in the liver implies the accumulation of particular lipid molecules such as diacylglycerol and ceramide, which in turn worsen insulin resistance [7–9]. Moreover, steatosis may induce chronic low-grade inflammation in the liver owing to immune cells infiltration and endoplasmic reticulum (ER) stress. When the liver develops overt inflammatory damage, NAFLD progresses to its advanced stage, nonalcoholic steatohepatitis (NASH) [10, 11]. NAFLD and NASH have been implicated as one of the most prevalent chronic liver diseases worldwide. However, the effective therapy for NASH is still not available [12, 13].

Recent clinical and experimental studies suggest that dietary supplementation of deep sea fish oil enriched in long-chain (LC) omega-3 PUFAs such as eicosapentaenoic acid (EPA) and docosahexaenoic acid (DHA) ameliorates obesity-associated NAFLD and NASH [14–18]. For instance, fish oil supplementation significantly reduces liver fat and aspartate aminotransferase levels in human subjects [14]. This antisteatotic effect of fish oil appears to be attributable to the inhibition of de novo lipogenesis and the activation of beta-oxidation, very-low density lipoproteins (VLDL) secretion, and bile acid synthesis [15]. Furthermore, fish oil possesses pleiotropic properties, which help attenuate hepatic steatosis-induced metaflammation and oxidative stress [17, 19].

Our previous transcriptomic study revealed that fish oil supplementation altered hepatic gene expression in a Western-style high-fat high-cholesterol diet- (WD-) induced rat model of NAFLD [20]. Specifically, LC n-3 PUFAs consumption significantly rescued the WD-induced dysregulation of hepatic expression of genes related to lipid metabolism and inflammation, such as* Srebf-1, Insig2, Abcg5/8, Mcp-1,* and* Socs1/2* [20]. Additionally, our study showed that fish oil restored the expression of core circadian clock genes such as* Per2, Per3,* and* Bmal1*, implying that fish oil may participate in the regulation of circadian clock rhythmicity [20]. How LC n-3 PUFAs alter hepatic gene expression remains to be explored. Several transcription factors such as peroxisome proliferator-activated receptor alpha (PPAR*α*), sterol regulatory element-binding protein 1/2 (SREBP1/2), carbohydrate response element-binding protein (ChREBP), and forkhead box O transcription factor 1 (FoxO1) are considered to account for fish oil regulation of hepatic gene transcription modification [21, 22]. Additionally, endogenous small noncoding RNAs known as microRNAs (miRNAs) may also be involved in high-fat diet- (HFD-) and/or obesity-induced dysregulation of hepatic gene expression, thereby bringing about NAFLD [23, 24]. In the present study, we conducted a RNA-sequencing (RNA-seq) analysis and investigated the roles of miRNAs in the effects of fish oil on protecting against WD-induced NAFLD.

## 2. Methods

### 2.1. Ethics, Consent, and Permissions

The animal experiments protocol was approved by Laboratory Animal Ethics Committee of Wuhan Polytechnic University (ID number: 20121009006). Animal care complied with institutional guidelines.

### 2.2. Animals

As described in a previous study [20], 9-week-old male Sprague–Dawley rats were randomly divided into three groups, control group (CON, *n* = 10), Western-style diet feeding group (WD, *n* = 10), and WD diet with fish oil feeding group (FOH, *n* = 10), and fed with a normal chow diet (10 kcal% fat), lard-rich WD diet (45 kcal% fat and 2% cholesterol, w/w), and fish oil rich diet (45 kcal% fat and 2% cholesterol, w/w; within 10% fish oil w/w), respectively. The diets were designed following the AIN-93 recommendations and the details were presented in the previous study. The animals were fed ad libitum for 16 weeks and then sacrificed for further studies.

### 2.3. Small RNA Library Construction and Sequencing

Total RNA was extracted from liver samples using a single-step acid guanidinium thiocyanate-phenol-chloroform method with minor modifications [25]. RNAs were examined for purity and quality using agarose gel electrophoresis and quantified using a SmartSpec (Bio-Rad) to determine the absorbance at A260. Aliquots of the total RNA (3 *μ*g) from 10 rats in each group were mixed together to generate the pooled sample and used for small RNA cDNA library preparation using a Balancer NGS Library Preparation Kit for small/microRNA (GnomeGen) following manufacturer's instruction. Briefly, RNAs were ligated to 3′ and 5′ adaptor sequentially. After reverse transcriptions, the cDNAs were used for PCR amplifications. Whole library was size fractionated by 10% native PAGE gel electrophoresis and bands corresponding to microRNA insertion were cut and eluted. After ethanol precipitation and washing, the purified small RNA libraries were quantified with Qubit Fluorometer (Life) and used for cluster generation and 36 nt single end sequencing analysis using the Illumina GAIIx (Illumina) according to the manufacturer's instructions.

### 2.4. Bioinformatic Analysis of Small RNA Sequences

Raw reads obtained from the libraries were first treated by Cutadapt and FASTX-toolkit to obtain the clean reads. The quality assessment and length distribution of sequencing data were assessed using FastQC software (http://www.bioinformatics.babraham.ac.uk/projects/fastqc/). Clean small RNA sequences longer than 12 nt from the libraries were aligned against Rfam database (Rfam 10.0) using Bowtie to identify the miRNA, lncRNA, sRNA, snRNA, snoRNA, tRNA, and others. The clean reads were then aligned against rat genome and mapped in rat microRNA database from miRBase 20 to identify the mature miRNAs and pre-miRNAs with hairpin structure [26].

### 2.5. MicroRNA Expression Analysis

The counts of conserved miRNAs identified in CON, WD, and FOH were used to calculate the TPM (Transcripts Per Million), and the differentially expressed microRNAs (DEMs) between FOH and WD groups were identified using edgeR [27]. The *P* values were calculated using negative binomial distribution and those miRNAs with fold change ≥ ±2 and *P* < 0.01 were termed as DEMs.

### 2.6. Prediction of DEM Target Gene

Both TargetScan (http://www.targetscan.org) [28] and miRanda (http://www.microrna.org/microrna/) [29] database were used to predict the potential target genes of resulting DEMs. The predicted targets were then applied for integrative analysis with previous mRNA expression profiling data, and those mRNAs negatively correlated with DEMs were considered as highly possible miRNA targets. The gene-gene network of these highly possible DEMs targets was analyzed using STRING (http://string-db.org).

### 2.7. Function Annotation of Predicted DEM Targets

The gene ontology (GO, http://geneontology.org/) analysis of predicted DEM target genes was conducted as previously described [30]. The terms with FDR < 0.05 were considered to be significant. A total of 3000 genes upon combining all TargetScan predicted results and miRanda predicted targets with top miRanda score of either FOH up- or downregulated miRNAs were annotated using Database for Annotation, Visualization and Integration Discovery (DAVID) [31] to further explore the function of miRNAs altered by fish oil.

### 2.8. Validation of Identified DEMs

To validate the small RNA-sequencing results, quantitative real-time RT-PCR (qRT-PCR) was performed to detect the expression of elected miRNAs. Total microRNAs from each rat in CON, WD, or FOH group were extracted using an EasyPure miRNA Kit (TransGen) according to manufacturer's instructions. Extracted miRNAs were then reversely transcribed and amplified using TransScript Green miRNA Two-Step qRT-PCR SuperMix (TransGen). Each qPCR sample was run in a total of 20 *μ*L volume and the thermocycling conditions were used: 120 s at 94°C and 45 cycles of 94°C for 5 s and 60°C for 30 s, followed by a melting ramp from 60 to 94°C. All reactions were performed in triplicate. U6 was used as the internal control. The primers used in qRT-PCR were listed in Supplementary Table  1 (see Supplementary Material available online at https://doi.org/10.1155/2017/2503847). The results were analyzed using 2^−ΔΔCt^ calculation. The data are presented as the means ± standard deviation (SD). The significance of differences in data between three groups was determined by one-way ANOVA analysis of variance followed by Student's *t*-test for equality of variances using SPSS 17.0 (IBM, USA). Differences were considered statistically significant at *P* < 0.05.

## 3. Results

### 3.1. Description of the Small RNA Libraries

In our previous study, we have illustrated that 16 weeks of fish oil feeding ameliorated WD diet-induced hyperlipidemia, hepatic steatosis, and inflammation, and the transcriptomic study has identified the differentially expressed hepatic mRNAs between the rats fed with WD diet or WD supplemented with fish oil (FOH) [20].

**Figure 1:**
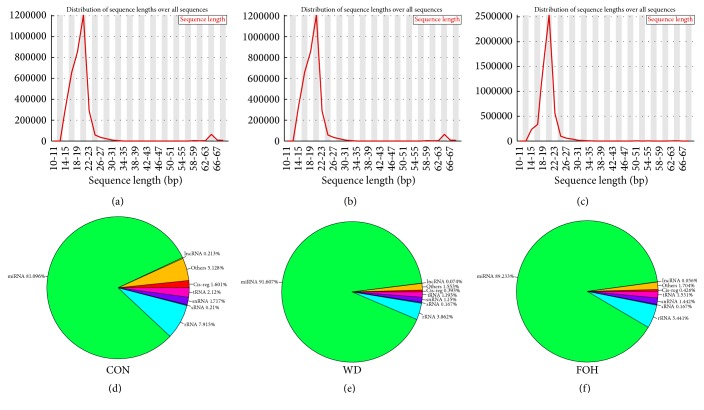
Genome-wide profiling of small RNAs in CON, WD, and FOH groups. (a)–(c) Frequency distribution of sequence lengths over all small RNA sequences in CON, WD, and FOH group, respectively. (d)–(f) Rfam classification of small RNAs in CON, WD, and FOH group, respectively.

In the present study, we extracted the small RNAs from livers of six rats in WD or FOH groups, respectively, and constructed the pooled small RNA libraries separately for WD and FOH to identify the DEMs. Also, the small RNAs from rats fed with normal lab chow were extracted to construct the control library (CON). A total of 7080492, 6519136, and 7509391 raw reads were obtained from the FOH, WD, and CON libraries, respectively, using the Illumina sequencing. After discarding reads with more than one undetected base “N,” adapters and adapter-adapter ligations, and other low-quality sequences, the remaining reads no shorter than 12 nt were set as clean reads. Finally, 5484790 (77.46%), 4874172 (74.77%), and 3607276 (48.04%) clean reads were obtained in FOH, WD, and CON library, respectively. The small RNA length distribution of libraries illustrated that the most abundant and diverse species were those at 20–22 nt in length, which is a typical size range for Dicer-derived products (Figures 1(a)–1(c)). These clean reads were aligned against Rfam database to identify the reads classification using Bowtie [32]. In all three groups, most of clean reads were identified as miRNAs (81.096%, 91.604%, and 89.233% in CON, WD, and FOH group, resp.), suggesting that the quality of three libraries was excellent, and the left reads were identified as rRNA, tRNA, snRNA, and so on (Figures 1(d)–1(f)).

### 3.2. Identification of Conserved miRNAs

The clean reads were then aligned against rat genome database. In FOH library, 5361730 (97.76%) reads were successfully mapped to rat genome with no more than one mismatch, and 4018402 (74.95%) of reads were uniquely mapped; in WD library, the mapped reads and uniquely mapped reads were 4752801 (97.51%) and 3651111 (76.82%), respectively; and in CON library, the mapped and uniquely mapped reads were 3463172 (96.01%) and 1935576 (55.89%), respectively. The mapped reads were selected to align against rat miRNA database in miRBase to identify conserved miRNAs and calculate the expression level. In FOH, WD, and CON groups, 3874946 (70.65%), 3578712 (73.42%), and 1870963 (51.87%) reads were annotated to known miRNA sequences, respectively. Those reads cannot match miRNAs which were aligned against rat pre-miRNA database, and in FOH, WD, and CON groups, a total of 151916, 134954, and 85457 reads were identified as pre-miRNA sequences, respectively.

### 3.3. Differentially Expressed miRNAs between FOH and WD Groups

**Figure 2:**
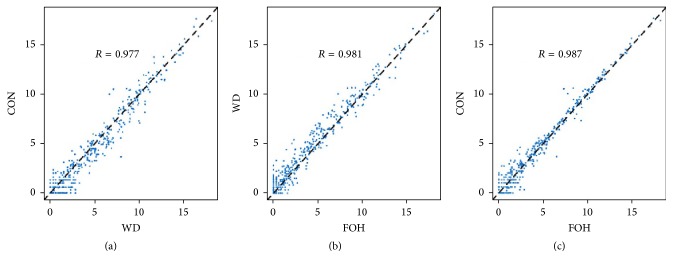
Scatter plot maps for miRNA expression levels between every two groups. Each plot represents an individual miRNA. (a) WD versus CON; (b) FOH versus WD; (c) FOH versus CON.

The transcripts per million (TPM) ((miRNA total reads/total clean reads) × 10^6^) value analysis was applied to normalize the expression of miRNAs and pre-miRNAs in different libraries. In FOH, WD, and CON groups, 405, 486, and 385 mature miRNAs were identified with TPM > 0, respectively (Supplemental Table 2). The expression levels of miRNAs expressed in all three libraries were quantified.  Figure 2 illustrated that most of identified miRNAs had similar expression patterns, and miRNA expression pattern in FOH group was slightly more correlative with the CON group compared with the WD group. The DEMs between FOH and WD groups were identified using edgeR. A total of 79 miRNAs were successfully identified as DEMs, among which 28 miRNAs were found to have a higher expression level and 51 miRNAs were downregulated in FOH group compared with WD group (Table 1).

### 3.4. Target Prediction of the DEMs

Normally, miRNAs regulate gene expression by binding the 5′ end 2–8 nt of miRNA seed region with 3′-UTR of target gene mRNA and inhibiting the target gene translation [33]. Nowadays, the target genes of most miRNAs have not been revealed exactly by biological investigation. Also, the functional analysis of DEMs depends on their target gene prediction by bioinformation approaches. In the current study, in order to investigate the functions of DEMs between FOH and WD groups, we combined the target gene prediction results of TargetScan and miRanda. A total of 4197 predicted genes with *P* < 0.005 (miRanda) or context score < −0.5 (TargetScan) were identified as the targets of FOH downregulated miRNAs, and 6405 genes were predicted as targets of FOH upregulated miRNAs (Supplemental Table 3).

### 3.5. Functional Categorization of Predicted Target Genes

A total of 10614 predicted targets of DEMs between FOH and WD groups were classified according to gene ontology (GO) annotation in* Rattus norvegicus* species. In the biological processes category, 5813 targets were mapped to reference list, and using Bonferroni correction, more than 800 GO terms were enriched with *P* < 0.05 (Supplemental Table 4). Unsurprisingly, several enriched terms with top fold enrichment were associated with metabolic processes, including regulation of lipid transport (GO:0032368, fold enrichment = 2.37, *P* = 1.10*E* − 04), cellular amino acid catabolic process (GO:0009063, fold = 2.39, *P* = 1.38*E* − 02), and carboxylic acid catabolic process (GO:0046395, fold = 2.1, *P* = 2.47*E* − 05). Additionally, some enriched terms were connected to protein expression, such as peptide secretion (GO:0002790, fold = 2.4, *P* = 1.69*E* − 02) and peptide transport (GO:0015833, fold = 2.28, *P* = 9.63*E* − 03); and some enriched terms implied that several critical signaling pathways were involved in DEMs target gene network, including regulation of calcium ion-dependent exocytosis (GO:0017158, fold = 2.27, *P* = 2.02*E* − 02), positive regulation of protein kinase B signaling (GO:0051897, fold = 2.25, *P* = 8.70*E* − 03), and stress-activated protein kinase signaling cascade (GO:0031098, fold = 2.21, *P* = 3.78*E* − 02). A series of hormone stimuli related terms were enriched, such as response to mineralocorticoid (GO:0051385, fold = 2.51, *P* = 4.62*E* − 03), cellular response to corticosteroid stimulus (GO:0071384, fold = 2.22, *P* = 6.26*E* − 04), cellular response to glucocorticoid stimulus (GO:0071385, fold = 2.22, *P* = 1.98*E* − 03), and response to insulin (GO:0032868, fold = 1.91, *P* = 1.34*E* − 06). Similarly, in the molecular function category, 162 terms were enriched with *P* < 0.05 (Supplemental Table 3), and the top fold enrichment terms included ionotropic glutamate receptor activity (GO:0004970), transmitter-gated channel activity (GO:0022835), excitatory extracellular ligand-gated ion channel activity (GO:0005231), vitamin binding (GO:0019842), calcium channel activity (GO:0005262), and hormone binding (GO:0042562). GO cellular component analysis revealed that most of predicted miRNA targets were enriched in cell projection cytoplasm (GO:0032838), transport vesicle membrane (GO:0030658), voltage-gated potassium channel complex (GO:0008076), and plasma membrane receptor complex (GO:0098802) (Supplementary Table 3). Interestingly, a number of terms related to neural functions were enriched in GO analysis, such as regulation of postsynaptic membrane potential (GO:0060078), dendrite morphogenesis (GO:0048813), neurotransmitter receptor activity (GO:0030594), dendritic spine (GO:0043197), and synaptic vesicle (GO:0008021). Whether the synapse and dendritic cells take part in DEMs-associated gene expression change in liver of fish oil fed NAFLD rats remains not clear and is worthy of further investigation.

To explore the roles of fish oil feeding-induced or inhibited miRNAs in NAFLD model rats, the predicted targets of up- and downregulated miRNAs were annotated using DAVID, respectively [31]. The GO, Kyoto Encyclopedia of Genes and Genomes (KEGG) pathway, and InterPro database were selected, and, due to DAVID limitation, a total of 3000 genes of TargetScan predicted targets and miRanda predicted targets with top miRanda score of either up- or downregulated miRNAs were chosen for DAVID analysis. The annotation analysis illustrated that the target genes of fish oil feeding downregulated miRNAs were mainly enriched in clusters associated with gene expression regulation, particularly positive regulation of transcription; ATP binding; vasculature development; protein kinase cascade, particularly MAPKKK cascade; cellular metabolism such as carboxylic acid binding and fatty acid binding; and ECM-receptor interaction. The predicted targets of FOH upregulated miRNAs were enriched in clusters including carboxylic acid and amino acid binding; ion homeostasis, particularly elevation of cytosolic calcium ion concentration; and membrane-bounded vesicle. Additionally, several clusters connected to neuron differentiation, synapse, or tube development were also enriched, which were consistent with the GO annotation results (Supplemental Table 5).

### 3.6. Integrative Analysis of DEMs and Differentially Expressed mRNAs

**Figure 3:**
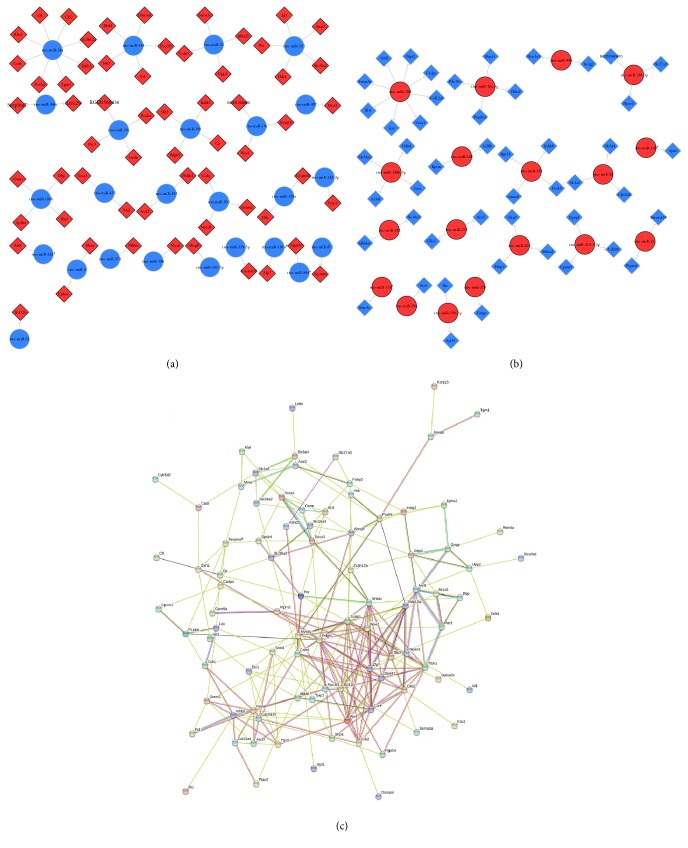
Integrative analysis of DEMs and their differentially expressed target mRNAs. Construction of functional networks of miRNA-mRNA between FOH and WD groups and the DEM target differentially expressed mRNAs. (a) FOH downregulated miRNAs and their targets; (b) FOH upregulated miRNAs and their targets. (c) Gene-gene network of DEMs target differentially expressed mRNAs.

In a previous study, we have identified the differentially expressed mRNAs between WD and FOH feeding animals using RNA-seq, and the results indicated that fish oil protects liver from WD-induced hepatic dyslipidemia and inflammation through altering the expression of a number of genes such as* Insig2, Abcg8, Pcsk9,* and* Per3*. The expression changes of these genes were validated using qRT-PCR [20]. However, how the fish oil feeding leads to these gene expression changes remains to be explored. Here we analyzed the predicted target genes of all DEMs which have been identified as differentially expressed mRNAs. We also examined whether the expression changes of differentially expressed miRNAs and mRNAs were negatively correlated, and whether the expression of these mRNAs was proposed to be inhibited, at least partly, by the identified miRNAs. The functional networks of miRNA-mRNA pairs were presented in Figure 3. The upregulation of 61 genes may be attributed to fish oil downregulated miRNAs (Figure 3(a)), as well as 48 genes which were identified as highly possible targets of FOH upregulated miRNAs (Figure 3(b)), and the gene-gene network of these DEMs target differentially expressed mRNAs was analyzed using STRING (10.0, Figure 3(c)). The findings illustrated that fish oil may restore the expression of circadian clock-related* Per3* via inhibiting the WD-induced overexpression of rno-miR-29c and that FOH upregulated the expression of rno-miR-328, possibly resulting in the inhibition of* Pcsk9*. Most DEMs appeared to have multiple targets. Among those DEMs, rno-miR-30d and rno-miR-34a were found to target 8 differentially expressed mRNAs, respectively.

### 3.7. Validation of Identified DEMs

**Figure 4:**
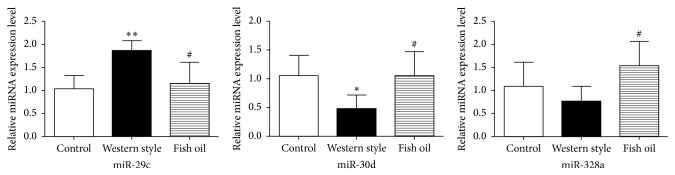
Expression of miRNAs by qRT-PCR. MiRNA expression of rno-miR-29c, rno-miR-328, and rno-miR-30d in WD, FOH, and CON groups was examined using qRT-PCR. Data are presented as means ± standard deviation, *n* = 10. *∗* and *∗∗*, WD versus CON; *P* < 0.05 and 0.01, respectively. #, FOH versus WD; *P* < 0.05.

The expression of rno-miR-29c, rno-miR-328, and rno-miR-30d in WD, FOH, and CON groups was further examined using qRT-PCR to validate the findings of comparative miRNA sequencing. The results showed that fish oil feeding inhibited the expression of rno-miR-29c and stimulated the expression of miR-30d and miR-328, where all were consistent with our miRNA transcriptomic results. Therefore, the present transcriptomic analyses were reliable (Figure 4).

## 4. Discussion

MicroRNAs play an epigenetic role in the regulation of gene expression. In metazoan such as rodent animals and human, mature miRNAs partially complementarily bind to target mRNAs and modestly repress and some particular miRNAs may induce [34] the gene expression [33]. Recent studies have indicated that miRNAs take part in the metabolic disorders such as NAFLD [23, 24, 35–39]. Several miRNAs, such as miR-122, miR-33a/b, and miR-34a, are involved in hepatic cholesterol and lipid homeostasis, and their circulating fragments are considered as biomarkers in NAFLD [23, 39–41]. Additionally, several high-throughput transcriptomic studies have identified the global miRNA expression changes in NAFLD animal models [42–46]. These findings suggest that the development of NAFLD, from steatosis to NASH, fibrosis/cirrhosis, and even hepatocellular carcinoma, is accompanied with the changes of miRNAs.

Chronic consumption of fish oil has shown to benefit human health [47]. For NAFLD, a well-organized meta-analysis study has revealed that the omega-3 PUFA supplementation can ameliorate the fat ectopic deposition in liver [14]. Indeed, numerous clinical trials and experimental studies have investigated the effects of fish oil on protecting against lipid metabolic disorder, insulin resistance, and inflammation in NAFLD [15, 18, 48]. Specifically, some studies have focused on the roles of miRNAs in the protective effects of fish oil or omega-3 PUFAs against metabolic syndrome and found that the intake of fish oil or DHA/EPA can modify the expression of miR-30b and miR-378 [49], miR-33a and miR-122 [50], miR-107 [51], miR-192, and miR-30c [52]. A recent clinical study investigated the circulating miRNAs changes of 192 common miRNAs in healthy women before and after an 8-week high PUFA diet intake and identified miR-106a, miR-130b, miR-221, and miR-221 which were correlated with diet-induced changes [53]. In addition, Zheng et al. investigated the roles of miRNA in omega-3 or omega-6 PUFAs feeding-induced inflammatory change in rats [54]. However, the genomic alteration of miRNA expression in fish oil-ameliorated fatty liver remains unclear. In the present study, we investigated the miRNA expression change in livers from fish oil fed NAFLD model rats. To our knowledge, this is the first study to explore the fish oil induced global hepatic miRNA expression change in WD-induced NAFLD rats.

In the current study, we successfully identified 79 DEMs between FOH and WD groups. Among these DEMs, the expression of rno-miR-33-5p and rno-miR-34a-5p was reduced in FOH compared with WD group. Rno-miR-33-5p is involved in the SREBP2 signaling pathway and plays an important role in cholesterol metabolic homeostasis, likely through target repressing the expression of ATP binding cassette transporter subfamily A, member 1 (ABCA1) [55]. ABCA1 is also targeted by miR-106b [56], which has been identified as another downregulated miRNA in FOH group in the present study. MiR-34a has been reported to regulate the expression of sirtuin 1 (SIRT1) in the liver, evidenced by the fact that the increased miR-34a levels in a high-fat and high calorie Western-style diet-induced obese mice led to reduced SIRT1 levels [57]. The functional roles of miR-33 and miR-34a in metabolic syndrome have been well reviewed [23]. In our study, fish oil feeding diminished the elevated rno-miR-33-5p and rno-miR-34a-5p expression levels in Western-style diet-induced NAFLD rats, indicating that these two miRNAs may contribute to the protective effects of fish oil on hepatic triglyceride and cholesterol metabolic disorder. Additionally, functional annotation of predicted target genes of DEMs implies that these DEMs may take part in the hepatic metabolic regulation of amino acids, lipids, and carboxylic acids. Moreover, some DEMs' targets are associated with cellular signaling transduction and response such as calcium ion signaling pathway and insulin signaling pathway and regulate gene expression on transcription and translation levels. These findings suggest that miRNAs are involved in the protective effects of fish oil against WD-induced metabolic disorder.

Integrative analysis of DEMs and differentially expressed mRNAs between FOH and WD groups helped identify the highly probable target genes of DEMs. Our findings indicated that fish oil feeding reduced the expression of rno-miR-29c and increased the expression of rno-miR-328 and rno-miR-30d in FOH group, and target regulated the expression of* Per3*,* Pcsk9*, and* Socs1*, respectively.* Per3* is a critical circadian rhythm gene and associated with obesity and diabetes [58].* Pcsk9* has attracted much attention for its crucial role in LDL receptor degradation and cholesterol metabolism [59].* Socs1* plays an important role in immune responses and takes part in insulin and leptin signaling pathways [60]. Our previous study has pointed out that fish oil feeding regulates the expression of these genes, and the present study revealed that the expression changes at least are partly attributed to miRNAs modification. The expression differences of* Lox, Insig2, Abcg8, Socs3*, and* Wee1* between FOH and WD groups may also be due to DEMs.

## 5. Conclusion

Our current study provides a prospective view of the roles of miRNAs in the protective effects of fish oil against Western-style diet-induced NAFLD. How fish oil regulates the particular miRNAs expression is worthy of further investigation.

## Supplementary Material

The supplementary files provided the useful information for readers. The description for each files have been listed in Supplemental Table 1. Primers for miRNA qRT-PCR. Supplemental Table 2. TPMs of identified miRNAs in CON, WD and FOH groups. Supplemental Table 3. Predicted target genes of identified DEMs. Supplemental Table 4. GO annotation of predicted targets of DEMs. Supplemental Table 5. DAVID analysis of predicted targets of DEMs.

## Figures and Tables

**Table 1:** Identified DEMs between FOH and WD groups.

FOH versus WD downregulated DEMs			FOH versus WD upregulated DEMs		
miRNA	Fold change	*P* value	miRNA	Fold change	*P* value
rno-let-7f-1-3p	0.18	4.13*E* − 05	rno-miR-100-5p	3.39	0
rno-miR-101a-5p	0.28	0.0059	rno-miR-10a-5p	2.58	0
rno-miR-106b-5p	0.25	8.44*E* − 88	rno-miR-1249	2.64	7.56*E* − 05
rno-miR-126b	0.31	1.90*E* − 23	rno-miR-139-3p	2.95	2.26*E* − 22
rno-miR-130a-3p	0.36	3.81*E* − 15	rno-miR-140-3p	2.15	3.72*E* − 261
rno-miR-142-3p	0.19	0	rno-miR-143-3p	2.24	0
rno-miR-142-5p	0.26	0	rno-miR-146b-5p	2.08	1.12*E* − 207
rno-miR-144-3p	0.35	0	rno-miR-148b-3p	3.31	0
rno-miR-144-5p	0.46	1.62*E* − 15	rno-miR-151-3p	2.70	3.70*E* − 176
rno-miR-146a-3p	0.33	4.00*E* − 19	rno-miR-151-5p	2.80	3.98*E* − 319
rno-miR-15b-3p	0.23	0.0041	rno-miR-152-5p	4.24	0.0044
rno-miR-17-5p	0.49	4.10*E* − 42	rno-miR-182	2.66	8.52*E* − 62
rno-miR-18a-5p	0.41	3.35*E* − 08	rno-miR-203b-3p	2.11	4.45*E* − 21
rno-miR-190a-5p	0.12	4.42*E* − 25	rno-miR-219-1-3p	6.66	4.92*E* − 05
rno-miR-193-3p	0.33	7.30*E* − 73	rno-miR-27b-5p	3.46	0.0037
rno-miR-19a-3p	0.32	1.39*E* − 38	rno-miR-28-5p	3.29	0
rno-miR-19b-3p	0.47	3.22*E* − 230	rno-miR-293-5p	6.05	0.0007
rno-miR-22-5p	0.41	1.11*E* − 13	rno-miR-30d-5p	2.05	0
rno-miR-223-3p	0.42	1.17*E* − 17	rno-miR-3102	2.69*E* + 09	0.0039
rno-miR-23a-5p	0.00	3.05*E* − 05	rno-miR-328a-3p	2.16	3.10*E* − 09
rno-miR-29b-3p	0.38	1.16*E* − 21	rno-miR-3586-3p	2.78	0
rno-miR-29c-3p	0.39	6.03*E* − 27	rno-miR-370-3p	3.42	6.46*E* − 06
rno-miR-301a-3p	0.00	4.77*E* − 07	rno-miR-375-3p	3.70	5.12*E* − 275
rno-miR-32-5p	0.28	1.69*E* − 38	rno-miR-425-5p	2.05	2.91*E* − 131
rno-miR-33-5p	0.13	3.64*E* − 19	rno-miR-598-3p	2.29	0.0004
rno-miR-330-5p	0.09	0.001	rno-miR-92a-3p	2.28	0
rno-miR-331-3p	0.44	0.0065	rno-miR-99a-5p	2.45	0
rno-miR-339-5p	0.44	7.48*E* − 112	rno-miR-99b-5p	5.41	0
rno-miR-345-5p	0.27	4.25*E* − 08			
rno-miR-34a-5p	0.46	4.92*E* − 39			
rno-miR-3556b	0.38	1.57*E* − 21			
rno-miR-3558-3p	0.00	0.0039			
rno-miR-3590-5p	0.36	7.46*E* − 15			
rno-miR-362-3p	0.43	2.26*E* − 35			
rno-miR-374-3p	0.43	3.91*E* − 05			
rno-miR-374-5p	0.30	3.24*E* − 142			
rno-miR-455-5p	0.49	3.53*E* − 06			
rno-miR-466c-5p	0.28	1.04*E* − 07			
rno-miR-490-3p	0.16	0.0023			
rno-miR-497-5p	0.38	2.81*E* − 72			
rno-miR-499-5p	0.39	2.15*E* − 17			
rno-miR-503-5p	0.41	5.64*E* − 08			
rno-miR-505-5p	0.00	0.0078125			
rno-miR-511-3p	0.42	2.86*E* − 21			
rno-miR-511-5p	0.00	0.002			
rno-miR-547-3p	0.04	6.94*E* − 08			
rno-miR-664-3p	0.36	4.11*E* − 32			
rno-miR-871-3p	0.00	0.0039			
rno-miR-872-5p	0.37	2.03*E* − 58			
rno-miR-99a-3p	0.11	2.46*E* − 13			
rno-miR-9a-5p	0.37	7.56*E* − 05			

## Abbreviations

NAFLD: Nonalcoholic fatty liver disease
PUFAs: n-3 polyunsaturated fatty acids
CON: Control diet
WD: A Western-style high-fat and high-cholesterol diet
FOH: A WD diet combined with fish oil
RT-PCR: Reverse transcription polymerase chain reaction
miRNA: MicroRNA
NASH: Nonalcoholic steatohepatitis
ERS: Endoplasmic reticulum stress
DEMs: Differentially expressed microRNAs
Srebf1: Sterol regulatory element-binding factor 1
PPAR*α*: Peroxisome proliferator-activated receptor alpha
GO: Gene ontology
SREBP1/2: Sterol regulatory element-binding protein 1/2
DAVID: Database for Annotation, Visualization and Integration Discovery
ChREBP: Carbohydrate response element-binding protein
FoxO1: Forkhead box O transcription factor 1
Socs2: Suppressor of cytokine signaling 2
Pers: Period genes
SIRT1: Sirtuin 1.

## Data Access

The full data sets have been submitted to NCBI Sequence Read Archive (SRA) under Accession SRA177759, Bioproject: PRJNA258329. All data generated or analyzed during the current study are available from the corresponding author on reasonable request.
